# The Role of Reproduction and Genetic Variation in Polish White-Backed Cows in the Breed Restoration Process

**DOI:** 10.3390/ani13172790

**Published:** 2023-09-02

**Authors:** Wioletta Sawicka-Zugaj, Witold Chabuz, Karolina Kasprzak-Filipek

**Affiliations:** Department of Cattle Breeding and Genetic Resources Conservation, University of Life Sciences in Lublin, 20-950 Lublin, Poland; wioletta.sawicka@up.lublin.pl (W.S.-Z.); karolina.kasprzak-filipek@up.lublin.pl (K.K.-F.)

**Keywords:** cattle reproduction, functional traits, cattle restoration

## Abstract

**Simple Summary:**

One of the few cattle breeds in the world undergoing restoration is the Polish White-Backed breed. The objective of this study was to use autosomal microsatellite markers to investigate genetic diversity in cows of this breed, taking into account their origin, and to evaluate their reproductive parameters. The findings of this work not only confirm the value of protecting local cattle breeds around the world, but may also be of importance in developing selection indices for highly productive breeds, in which reproductive functioning should be one of the most important factors considered.

**Abstract:**

Local breeds are the main reservoir of biodiversity of farm animals. According to FAO, they account for 87% of all described breeds in the world. To ensure that they are adequately protected, they should be continually monitored for genetic variation. Another crucial factor is reproduction, which is the most important guarantee of population growth. In the present study, genetic variation in 372 Polish White-Backed cows was determined using DNA microsatellite sequences, taking into account their parentage. Reproductive parameters were analysed as well, based on data from 3658 lactations of 1128 Polish White-Backed cows. The results indicate that despite the small initial population and the implementation of a moderate selection of animals, the existing population of Polish White-Backed cattle has a high level of genetic variation, reflected in the degree of heterozygosity (0.761). Regarding reproductive traits, despite their late age at first calving, Polish White-Backed cows were shown to be distinguished by very good fertility parameters in comparison to other breeds raised in Poland. These findings not only confirm the value of protecting local cattle breeds around the world but may also be of importance in developing selection indices for highly productive breeds, in which reproductive functioning should be one of the most important factors considered.

## 1. Introduction

Restoration and conservation of local livestock breeds possessing unique traits is of importance not only for agriculture and global food security but also for rural development, nature conservation, cultural heritage, and science [1]. The most valued traits in farm animals include longevity, good health, fertility, prolificacy, high product quality, and high genetic variation [2,3]. Unfortunately, these traits have been reduced in highly productive breeds by selection for high productivity. Intensive breeding selection has led to a decline in genetic variation and to inbreeding depression. This has entailed many unfavourable changes associated with a decline in the vigour and fertility of animals, and leading to a reduction in their reproductive capacity, health, and survival rates [4]. Furthermore, an increase in relatedness has been confirmed to increase calf mortality and delay growth, and thus prolong the time that animals need to reach sexual maturity, and also increase the risk of culling and loss of replacement heifers before their first calving [5]. A high level of inbreeding also weakens the population due to an increase in the degree of homozygosity and in the expression of harmful recessive alleles [6].

The Food and Agriculture Organization (FAO) reports that among 8190 breeds of farm animals, 87% are local breeds and 1984 breeds are endangered [7]. According to Gandini et al. [8], there are several reasons for the marginalization of native breeds: breeding to produce more productive animals, the development of intensive production systems, human population growth and increased demand for food, globalization, breeding and biotechnological revolutions, and climate change. The development of reproductive biotechnology and of animal feeding and production technology has led to a significant decline in the use of local breeds of farm animals all over the world. This valuable reservoir of unique genes has come under the threat of extinction, although studies [9,10] confirm that the genetic diversity of native breeds may play a major role in future breeding (due to changes in market demands, new diseases, or climate change).

One of the oldest cattle breeds in Poland is the dual-purpose (milk and beef) White-Backed breed. According to the FAO [7], this breed is currently classified as an endangered population. Due to ‘Holsteinization’, it was recognized as extinct in the mid-20th century. A few individuals (about 50–100) were encountered in eastern Poland, providing the basis for initiating the restoration process. Its restoration and inclusion in a genetic resources conservation programme began in the early 2000s, and, in consequence, the current population of cows of this breed entered in breeding books numbers is 970. According to the principles of breed restoration, animals of undocumented origin can be registered in breed books if they conform to the breed standard. Such individuals currently account for about 30% of the White-Backed population. In addition to the rare coat colour (side colouring) of the Polish White-Backed breed, research by Barłowska et al. [11] and Litwińczuk et al. [12] has shown that their milk has favourable physical and chemical characteristics, especially for processing. Similar conclusions were drawn from an analysis of the physical and chemical properties of the meat of Polish White-Backed cattle [13]. Their high fattening capacity has been observed in particular on semi-intensive farms using on-farm feed from permanent grassland [14]. These cattle are also highly effective in active nature conservation owing to their beneficial impact on the species diversity of plants, as demonstrated in a study by Rysiak et al. [15] carried out in the buffer zone of Polesie National Park. These are some of the major arguments in favour of continual monitoring and recording of changes taking place in the Polish White-Backed cattle population.

Changes in genetic variation can be monitored owing to the development of molecular genetic analysis at the level of deoxyribonucleic acid (DNA), using proven and widely used genetic markers. Demonstration of genetic diversity and population structure is also of great importance for the sustainable use of genetic resources. Widely used markers in genetic analysis of populations of farm animals are DNA microsatellite sequences, regarded as an ideal tool due to traits, such as numerous repetitions throughout the euchromatic region of the genome, codominance, and high polymorphism [16,17]. Their use is widespread—for assessment of genetic diversity [17], genetic distance [2], or relationships between breeds of farm animals [18], as well as for confirmation of origin [19]. 

In addition to a high level of genetic variation, reproductive parameters are an important factor determining both breeding success and economic success in livestock farming [20]. These depend on numerous factors, such as diet, the season of parturition, welfare conditions, and intensity of production [21,22]. Low fertility—specifically additional insemination procedures, veterinary services, and increased culling—can increase the cost of herd maintenance [23]. Highly productive cows are characterized by low fertility, which prolongs the calving interval and at the same time significantly shortens the animals’ productive life [24].

The objective of the study was to use autosomal microsatellite markers to investigate genetic diversity in Polish White-Backed cows, taking into account their origin, and to evaluate their reproductive parameters.

## 2. Materials and Methods

### 2.1. Genetic Analysis

The material for the study consisted of hair bulbs from 372 Polish White-Backed cows protected by a genetic resources conservation programme, selected on the basis of parentage. The hair bulbs were collected for the purposes of breeding work. The cows selected for the study were unrelated, going back two generations. The animals were divided into three groups: G1—cows with no documented origin (178); G2—cows with documentation of the either the mother or the father (86); and G3—cows with documentation of both parents and grandparents of the Polish White-Backed breed (108).

DNA was isolated using a commercial kit for isolation of nucleic acids from biological traces (Sherlock AX A&A Biotechnology), according to the procedure described by the manufacturer. DNA was amplified in a multiplex reaction (12 loci) using the Bovine Genotypes Panel 1.2 Kit (ThermoFisher Scientific, Waltham, MA, USA), containing 12 markers from the ISAG-recommended STR panel (BM1818, BM1824, BM2113, ETH3, ETH10, ETH225, INRA23, SPS115, TGLA53, TGLA122, TGLA126, and TGLA227).

Microsatellite sequences were amplified according to the manufacturer’s protocol, using 12 primer pairs labelled with four fluorescent dyes. PCR products were analysed by capillary electrophoresis in the 3130xl genetic analyser (Applied Biosystems, Waltham, MA, USA) and separated using the GeneScan-500 LIZ Size Standard (Applied Biosystems). The electrophoretic separation results were analysed using GeneMapper 4.0 software (Applied Biosystems). 

### 2.2. Analysis of Reproduction

The analysis included the entire population of Polish White-Backed cows covered by the genetic resources conservation programme during the period from 2003 (when breed restoration was initiated) to 2022. In total, 3658 lactations of 1128 cows were included in the analysis, including 372 individuals analyzed for genetic variation. 

Information obtained from milk performance evaluation of Polish White-Backed cows conducted by the Polish Federation of Cattle Breeders and Dairy Farmers was grouped according to genotype (R1—cows with no documented origin; R2—cows with documentation of the either the mother or the father; and R3—cows with documentation of parents and grandparents of the Polish White-Backed breed) and age at first calving (early—up to 24 months; intermediate—from 24.1 to 26 months; and late—26.1 months and later).

The following reproductive parameters were determined for the groups of animals: age at first calving, days open (number of days from calving to conception), calving interval (number of days from calving to calving), dry period (number of days from last milking to calving), calving-to-first-service interval (number of days between calving and the first service), service period (number of days between the first service and conception), services per conception (number of insemination procedures per conception), and gestation length. 

### 2.3. Statistical Analysis

Basic parameters of genetic variation, i.e., observed number of alleles (No) and effective number of alleles (Ne), were estimated for each locus and population using GenAIEx 6.5 software [25]. Observed heterozygosity (Ho), expected heterozygosity (He), average heterozygosity (Ha), and the fixation index (Fis) were calculated using CERVUS 2.0 software [26]. The Hardy–Weinberg equilibrium (HWE) was determined by the chi^2^ test, using GENEPOP 4.0 software [27].

The information obtained from the reproductive performance evaluation of Polish White-Backed cows was used to create a database. Statistical analysis of the database was performed by one-way analysis of variance (ANOVA) of means for each parameter using StatSoft Inc. STATISTICA 9.0 software. The groups had a normal distribution. Significance of differences between values for groups was determined by Tukey’s test. Means designated a,b differ significantly at *p* ≤ 0.05; means designated A,B differ significantly at *p* ≤ 0.01.

## 3. Results

### 3.1. Results of Genetic Variation Analysis

The results of the analysis of DNA microsatellite sequences are presented in Table 1. Among 372 samples analysed, a total of 382 different alleles were identified in the 12 microsatellite sequences. Differences were observed in the average number of alleles per locus between groups of animals. It was highest in the group of animals of unknown origin (12.167), intermediate in the group of cows with one known parent (11.000), and lowest in the population with known parents and grandparents (8.667). In all three groups, differences were noted in the number of alleles identified in individual microsatellite loci, except for SPS115 and TGLA126, with seven and five, respectively, in all three groups. In addition, the number of alleles in these two loci was the lowest among all those analysed in the three groups. The locus with the highest number of alleles was also different in each group: locus BM2113 in G1 (18 alleles), INRA23 in G2 (18 alleles), and TGLA53 in G3 (14 alleles). In the case of the effective number of alleles (Ne), groups G1 and G2 were shown to differ only slightly (6.450 and 6.569), while the value was much lower in G3 (4.472). In each of the groups, a substantial majority of microsatellite loci deviated from the Hardy–Weinberg equilibrium (HWE) (at *p* < 0.01), except for loci ETH10, TGLA126, and TGLA227 in G1, loci ETH10, SPS115, TGLA53, and TGLA126 in G2, and loci ETH3, SPS115, TGLA53, TGLA126, and TGLA227 in G3. The average heterozygosity was high in all groups (>0.7). Observed heterozygosity was lowest in the animals with one known parent (G2 (0.702)) and highest in those of known origin for two generations (G3 (0.755)). The locus with the highest observed heterozygosity was BM2113 in group G3 (0.926), while locus TGLA126 had the lowest Ho values in both G2 and G1 (0.581 and 0.595, respectively). The estimated value of Fis, which describes an excess or deficiency of heterozygotes in the population, ranged from −0.110 (BM2113) in G3 to 0.261 (ETH225) in G2. The average value for this parameter was 0.00 in the group of cows of fully known origin, 0.096 in G1, and 0.131 in G2.

### 3.2. Results of Reproduction Analysis

Indicators of fertility were determined in each group of Polish White-Backed cattle: age at first calving, calving interval, days open, calving-to-first-service interval, service period, services per conception, dry period length, and gestation length. The data in Table 2 show significant relationships between these fertility parameters and the animals’ genotype. Cows with a fully known origin generally had more favorable reproductive parameters than those of unknown origin. The following periods were statistically significantly (*p* < 0.01) shorter in that group than in group R1: calving interval by 16.57 days and days open by 16.15. In addition, in the R3 group, significantly fewer services per conception (1.59) were found in relation to the R2 group (2.01) and the R1 group (1.96), which significantly shortened the period of first service to conception. A different tendency was noted for age at first calving, which was earliest for heifers from group R1 (unknown origin), at 811.43 days, and latest for those with one known parent (R2), at 870.69 days. Cows from group R3 (fully known origin) first calved at the age of 850.35 days. The differences between means were confirmed statistically (*p* < 0.01). The average milk yield for 305-day lactation in the cows ranged from 4332.26 kg of milk in R1 to 4642.38 kg in R3. The differences in milk yield were not statistically significant.

## 4. Discussion

In the present study, to describe generational changes in the genetic variation of Polish White-Backed cows, a tested and reliable genetic tool was used—DNA microsatellite sequences. The high average number of alleles per locus in all analysed groups of Polish White-Backed cattle indicates high polymorphism of the microsatellite sequences used and high genetic diversity in the population. A study by Brasil et al. [29] conducted in nine Brazilian cattle breeds using 11 microsatellite sequences showed an equally high average number of alleles per locus (13.1) as the present study. A much higher value (16.36) was reported in an analysis of four Algerian cattle breeds, based on 22 microsatellite sequences [2], and a much lower value (6.72) in an assessment of the genetic variation of Italian and Croatian breeds [30]. The effective number of alleles can be observed to be higher in Algerian breeds (7.17) [2] than in the present study, while in the Lebedyn breed, it was shown to be much lower (3.208) in an analysis based on 10 microsatellite sequences [31]. 

However, a decline of over 28% was noted in the average number of alleles per locus in the group of cows of known origin for two generations relative to the group of animals of unknown origin. The influence of breeding selection on the degree of genetic variation in a breed is well known [32]. Polish White-Backed cattle are not subject to such rigorous selection by breeders as highly productive breeds, such as Holstein-Friesian; however, once breeding books for this breed have been opened outside the genetic resources conservation programme, there is a breeding programme carried out with moderate selection. Deviation from the Hardy–Weinberg equilibrium (HWE) in a large group of analysed microsatellite loci can have a number of causes. The most important include genetic drift, the presence of null alleles, and selection [33].

Our study showed a high level of expected heterozygosity (>0.7). A similar level was noted in local Senegalese breeds of zebu cattle, ranging from 0.730 to 0.799 [34], in local Algerian breeds, at 0.84 [2], and in Lithuanian dairy breeds, i.e., Lithuanian Red cattle and Lithuanian Red-and-White cattle, at 0.712 and 0.732, respectively [35]. Much lower values were obtained in studies on Lebedyn cattle (0.670) [31], Taro white cattle (0.628) [36], and the popular Holstein-Friesian breed (0.699) [37]. The observed heterozygosity values in the White-Backed population were also high (above 0.7). Equally high values were obtained for the local Syrian breed Shami (0.730) [37] and for local Lithuanian breeds, namely, Black and White (0.743), Red (0.705), and Red and White (0.724) [35]. The results of the present study and the literature results may indicate a high degree of genetic variation in indigenous breeds around the world. 

The Fis inbreeding coefficient is a parameter indicating a reduction in heterozygotes due to non-random mating [38]. In the case of Fis > 0, there is a deficiency of heterozygous individuals, while Fis < 0 indicates an excess of heterozygotes. In the present study, the average Fis values were slightly above 0, ranging from 0.000 in G3 to 0.131 in G2. The results are markedly lower than those reported for other European breeds [30,39,40]. 

An important reference point for the results pertaining to genetic variation obtained in the present study is a paper by Sawicka-Zugaj et al. [41] presenting an assessment of the reproductive performance and genetic variation of Polish White-Backed bulls, which showed a high level of heterozygosity (0.7597) and high Fis (−0.0587). The authors noted that this effect was obtained using an appropriate breed restoration plan, and, in particular, application of the principle of leaving only one son from the father for breeding.

In the case of intensive milk production, the optimal age at first calving for maximizing the yield and the productive life of cows in herds is considered to be 24–27 months [42]. In the present study of cows of the Polish White-Backed breed, which is used for both milk and meat, the average age at first calving was 28 months. A similar age—27.64 months—was reported by Gandini et al. [43] for the local Italian breed Reggiana. This indicates that age at first calving is later in indigenous breeds. This is not usually associated with milk yield but with how reproduction is organized in herds. In highly productive cattle breeds, such as Holstein-Friesian, the age at first calving is somewhat earlier—about 26 months [44]. However, at a time when efforts are made to continually increase the intensification of production, it is believed that costs can be reduced by as much as 18% when the age at first calving is reduced to 21 months [45,46]. 

A study by Bieber et al. [47] showed that European local cattle breeds have a shorter calving interval, fewer days open, and fewer services per conception than highly productive commercial breeds. Comparison of the results obtained for Polish White-Backed cows with the results for Polish Holstein-Friesians reported by Bieber et al. [47] reveals that the calving interval was much shorter in the present study, while the number of services per conception and number of days open were slightly higher. Organized breeding of Polish White-Backed cows of fully known origin (R3) has improved all of the analysed fertility parameters, in particular the calving interval, the number of services per conception, and, thus, the shorter period of first service to conception and the days open. This is the positive effect of 20 years of well-conducted breeding work in the restored breed.

Evaluation of the milk and reproductive performance of cattle in Poland, carried out by the Polish Federation of Cattle Breeders and Dairy Farmers [48], showed that for the entire population of dairy cattle in Poland, of which 96.52% are Polish Holstein-Friesian cows, the average age at first calving was 800 days (26.67 months), and the average calving interval was 426 days. It is worth noting that the age at first calving was earliest for the Jersey (795 days) and Polish Black-and-White Holstein-Friesian (796 days) breeds and latest for the Polish Black-and-White (908 days) and Polish White-Backed (889 days) breeds. Given that both the Polish Black-and-White and Polish White-Backed breeds are local breeds, a later age at first calving can be considered a characteristic trait of native, extensively used breeds. It is worth noting that the report shows that in 2021, Polish White-Backed cows had the shortest calving interval (404 days) among all dairy cow populations in Poland.

## 5. Conclusions

Twenty years after the start of the Polish White-Backed breed restoration programme and the implementation of moderate selection of animals, assessment of genetic variation showed that its level was high in the group of animals of fully known origin. The high degree of heterozygosity and the inbreeding coefficient (Fis) of 0 indicate that in situ conservation of the Polish White-Backed breed was successful. Moreover, well conducted breeding work in the breed restoration process resulted in favourable reproductive parameters, i.e., a much shorter calving interval, fewer services per conception, and, in consequence, shorter periods from first service to conception and days open. 

The results confirm the need for continuous monitoring of indigenous breeds, for both genetic variation and production parameters, and, at the same time, can provide inspiration and hope for organizations deciding to fight for the survival of local breeds of farm animals—not only of cattle.

## Figures and Tables

**Table 1:** Measures of genetic variation in the analysed populations.

Locus	G1						G2						G3						Ha
	*N* = 178						*N* = 86						*N* = 108						
	Na	Ne	HWE	Ho	He	Fis	Na	Ne	HWE	Ho	He	Fis	Na	Ne	HWE	Ho	He	Fis	
BM1818	10	5.028	***	0.697	0.806	0.130	10	5.974	*	0.651	0.842	0.218	7	3.050	**	0.667	0.678	0.008	0.769
BM1824	10	6.477	***	0.685	0.850	0.189	8	6.951	***	0.744	0.866	0.131	6	3.812	***	0.630	0.745	0.146	0.813
BM2113	18	10.561	***	0.764	0.910	0.156	14	10.215	**	0.767	0.913	0.149	10	6.037	***	0.926	0.842	−0.110	0.881
ETH3	10	6.171	***	0.742	0.843	0.115	10	4.859	***	0.651	0.804	0.180	8	3.104	NS	0.593	0.684	0.126	0.770
ETH10	8	4.035	NS	0.730	0.756	0.029	9	4.931	NS	0.721	0.807	0.096	9	3.978	***	0.741	0.756	0.010	0.766
ETH225	15	6.876	***	0.798	0.859	0.066	14	8.443	***	0.651	0.892	0.261	8	4.567	***	0.796	0.788	−0.019	0.839
INRA23	15	7.375	***	0.685	0.869	0.207	18	10.446	***	0.721	0.915	0.203	9	5.385	***	0.796	0.822	0.022	0.861
SPS115	7	2.535	***	0.618	0.609	−0.020	7	2.664	NS	0.605	0.632	0.032	7	2.663	NS	0.667	0.630	−0.067	0.618
TGLA53	17	9.464	**	0.764	0.899	0.146	15	8.805	NS	0.907	0.897	−0.023	14	7.209	NS	0.852	0.869	0.011	0.881
TGLA122	17	8.395	***	0.798	0.886	0.094	12	5.569	***	0.721	0.830	0.121	12	5.134	***	0.870	0.813	−0.081	0.835
TGLA126	5	2.618	NS	0.595	0.625	0.036	5	2.438	NS	0.581	0.597	0.014	5	2.988	NS	0.722	0.671	−0.086	0.624
TGLA227	14	7.870	NS	0.865	0.878	0.009	10	7.532	**	0.698	0.877	0.195	9	5.740	NS	0.796	0.833	0.036	0.855
Mean ± SD	12.167 ± 4.366	6.450 ± 2.536		0.7285 ± 0.077	0.816 ± 0.102	0.096	11.000 ± 3.717	6.569 ± 2.649		0.702 ± 0.0858	0.823 ± 0.105	0.131	8.667 ± 2.498	4.472 ± 1.442		0.755 ± 0.103	0.761 ± 0.079	0.000	0.793 ± 0.090

G1—population of unknown origin; G2—population with one known parent; G3—population of known origin for 2 generations; N—number of individuals in group; Na—number of alleles in locus; Ne—effective number of alleles; HWE—chi-square values of test for HWE; NS: *p* > 0.05—not significant; * *p* < 0.05—significant; ** *p* < 0.01—highly significant; *** *p* < 0.001—very highly significant; Ho—observed heterozygosity; He—expected heterozygosity; Ha—average heterozygosity; Fis—Wright’s [28] fixation index.

**Table 2:** Statistical analysis of reproductive traits in groups of Polish White-Backed cows.

	R1		R2		R3		Total	
	X	SD	X	SD	X	SD	X	SD
Number of lactations	1420		600		1638		3658	
Age at first calving (days and months)	811.43 A		870.69 B		850.35 C		844.53	
	(27.05)	139.87	(29.02)	133.67	(28.35)	124.07	(28.15)	131.61
Calving interval (days)	409.98 A	92.33	410.69 A	100.75	393.41 B	78.75	402.68	88.41
Days open (days)	129.03 A	92.23	129.28 A	100.89	112.88 B	78.75	121.84	88.37
Calving to first service (days)	81.91	54.78	80.33	52.10	79.12	48.01	80.40	51.40
First service to conception (days)	46.89 A	72.45	48.84 A	84.37	33.55 B	62.36	41.24	70.65
Services per conception	1.96 A	1.18	2.01 A	1.40	1.59 B	0.82	1.80	1.09
Dry period (days)	85.94 A	69.12	85.00	71.57	78.89 B	62.07	82.81	67.09
Gestation length (days) 305-day milk production (kg)	280.96	5.41	281.74	5.92	280.30	5.93	280.78	5.76
	4332.26	843.45	4381.02	1024.36	4642.38	815.21	4461.64	912.07

R1—population of unknown origin; R2—population with one known parent; R3—population of known origin for 2 generations; A–C—means in rows with different letters differ significantly at *p* ≤ 0.01.

## Data Availability

The datasets used and/or analyzed during the current study are available from the corresponding author upon reasonable request.

## References

[1] Sponenberg D.P., Beranger J., Martin A.M., Couch C.R. (2018). Conservation of rare and local breeds of livestock. Rev. Sci. Technol. Off. Int. Epizoot..

[2] Rahal O., Aissaoui C., Ata N., Yilmaz O., Cemal I., Ameur Ameur A., Gaouar S.B.S. (2021). Genetic characterization of four Algerian cattle breeds using microsatellite markers. Anim. Biotechnol..

[3] Suprovych T.M., Suprovych M.P., Mokhnachova N.B., Biriukova O.D., Strojanovska L.V., Chepurna V.A. (2021). Genetic variability and biodiversity of Ukrainian Gray cattle by the BoLA-DRB3 gene. Regul. Mech. Biosyst..

[4] Bjelland D.W., Weigel K.A., Vukasinovic N., Nkrumah J.D. (2013). Evaluation of inbreeding depression in Holstein cattle using whole-genome SNP markers and alternative measures of genomic inbreeding. J. Dairy Sci..

[5] Cassell B.G. (1999). Effect of Inbreeding on Lifetime Performance of Dairy Cows. Adv. Dairy Technol..

[6] Hofmanová B., Vostrý L., Vostrá-Vydrová H., Dokoupilová A., Majzlík I. (2019). Estimation of genetic and non-genetic effects influencing coat colour in black horses. Czech J. Anim. Sci..

[7] Domestic Animal Diversity Information System (DAD-IS). http://www.fao.org/dad-is/en/.

[8] Gandini G., Avon L., Bohte-Wilhelmus D., Bay E., Colinet F.G., Choroszy Z., Díaz C., Duclos D., Fernández J., Gengler N. (2010). Motives and values in farming local cattle breeds in Europe: A survey on 15 breeds. Anim. Genet. Resour..

[9] Bruford M.W., Bradley D.G., Luikart G. (2003). DNA markers reveal the complexity of livestock domestication. Nat. Rev. Genet..

[10] Toro M.A., Fernández J., Caballero A. (2009). Molecular characterization of breeds and its use in conservation. Livest. Sci..

[11] Barłowska J., Szwajkowska M., Litwińczuk Z., Król J. (2011). Nutritional value and technological suitability of milk from various animal species used for dairy production. Compr. Rev. Food Sci. Food Saf..

[12] Litwińczuk Z., Barłowska J., Chabuz W., Brodziak A. (2012). Nutritional value and technological suitability of milk from cows of three Polish breeds included in the genetic resources conservation programme. Ann. Anim. Sci..

[13] Litwińczuk Z., Florek M., Domaradzki P., Żółkiewski P. (2014). Physicochemical properties of meat from young bulls of 3 native breeds: Polish red, white-backed, and Polish black-and-white, as well as of simmental and Polish holstein-fresian breeds. Food. Sci. Technol. Qual..

[14] Litwińczuk Z., Żółkiewski P., Florek M., Chabuz W., Domaradzki P. (2014). Semi-intensive fattening suitability and slaughter value of young bulls of three Polish native breeds in comparison with Polish Holstein-Friesian and Simmental. Ann. Anim. Sci..

[15] Rysiak A., Chabuz W., Sawicka-Zugaj W., Jan Zdulski Grzywaczewski G., Kulik M. (2021). Comparative impacts of grazing and mowing on the floristics of grasslands in the buffer zone of Polesie National Park, eastern Poland. Glob. Ecol. Conserv..

[16] Bennett P. (2000). Demystified … Microsatellites. J. Clin. Pathol. Mol. Pathol..

[17] Agung P.P., Saputra F., Zein M.S.A., Wulandari A.S., Putra W.P.B., Said S., Jakaria J. (2019). Genetic diversity of Indonesian cattle breeds based on microsatellite markers. Asian-Australas. J. Anim. Sci..

[18] Karsli T., Demir E., Fidan H.G., Aslan M., Karsli B.A., Arik I.Z., Semerci E.S., Karabag K., Balcioglu M.S. (2020). Determination of genetic variability, population structure and genetic differentiation of indigenous Turkish goat breeds based on SSR loci. Small Rumin. Res..

[19] Yu G.C., Tang Q.Z., Long K.R., Che T.D., Li M.Z., Shuai S.R. (2015). Effectiveness of microsatellite and single nucleotide polymorphism markers for parentage analysis in European domestic pigs. Genet. Mol. Res..

[20] Abraham F. (2017). An Overview on Functional Causes of Infertility in cows. JFIV Reprod. Med. Genet..

[21] Riecka Z., Candrák J., Strapáková E. (2010). Factors Affecting Holstein Cattle Fertility Traits in the Slovak Republic. Anim. Sci. Biotechnol..

[22] Berry D.P., Friggens N.C., Lucy M., Roche J.R. (2016). Milk Production and Fertility in Cattle. Annu. Rev. Anim. Biosci..

[23] Kumar A., Mandal A., Gupta A.K., Karunakaran M., Das S.K., Dutta T.K. (2018). Genetic analysis of fertility traits in Jersey crossbred cows. Indian J. Anim. Res..

[24] Gonzalez-Recio O., Alenda R., Chang Y.M., Weigel K.A., Gianola D. (2006). Selection for female fertility using censored fertility traits and investigation of the relationship with milk production. J. Dairy Sci..

[25] Peakall R., Smouse P.E. (2012). GenAlEx 6.5: Genetic analysis in Excel. Population genetic software for teaching and research–an update. Bioinformatics.

[26] Marshall T.C., Slate J., Kruuk L.E.B., Pemberton J.M. (1998). Statistical confidence for likelihood-based paternity inference in natural populations. Mol. Ecol..

[27] Rousset F. (2008). GENEPOP’007: A complete re-implementation of the genepop software for Windows and Linux. Mol. Ecol. Resour..

[28] Wright S. (1978). Evolution and the Genetics of Populations: Variability within and among Natural Populations.

[29] Brasil B.S.A.F., Coelho E.G.A., Drummond M.G., Oliveira D.A.A. (2013). Genetic diversity and differentiation of exotic and American commercial cattle breeds raised in Brazil. Genet. Mol. Res..

[30] Maretto F., Ramljak J., Sbarra F., Penasa M., Mantovani R., Ivanković A., Bittante G. (2012). Genetic relationships among Italian and Croatian Podolian cattle breeds assessed by microsatellite markers. Livest. Sci..

[31] Ladyka V.I., Khmelnychyi L.M., Lyashenko Y.V., Kulibaba R.O. (2019). Analysis of the genetic structure of a population of Lebedyn cattle by microsatellite markers. Regul. Mech. Biosyst..

[32] Macedo F.L., Christensen O.F., Legarra A. (2021). Selection and drift reduce genetic variation for milk yield in Manech Tête Rousse dairy sheep. JDS Commun..

[33] Chen B., Cole J.W., Grond-Ginsbach C. (2017). Departure from Hardy Weinberg Equilibrium and Genotyping Error. Front. Genet..

[34] Ndiaye N.P., Sow A., Dayo G.K., Ndiaye S., Sawadogo G.J., Sembène M. (2015). Genetic diversity and phylogenetic relationships in local cattle breeds of Senegal based on autosomal microsatellite markers. Vet. World.

[35] Lapickis R., Griciuvienė L., Aleksandravičienė A., Lipatova I., Paulauskas A. (2021). Genetic variability of dairy cattle breeds in Lithuania. Biologija.

[36] Heryani L.G.S.S., Wandia I.N., Suarna I.W., Puja I.K., Susari N.N.W., Agustina K.K. (2019). Short communication: Molecular characteristic of taro white cattle based on DNA microsatellite markers. Biodiversitas.

[37] Alsalh M.A., Bakai A., Feyzullaev F.R., Bakai F.R., Lepekhina T.V., Mkrtchyan G., Krovikova A., Mekhtieva K., Alyaseen O.A. (2021). Comparative Characteristics of the Genetic Structure of the Syrian Cattle Breed Compared to Holstein and Aberdeen-Angus Breeds. J. Adv. Vet. Anim. Res..

[38] Hedrick P.W., Kalinowski S.T. (2000). Inbreeding Depression in Conservation Biology. Annu. Rev. Ecol. Evol. Syst..

[39] Cecchi F., Ciampolini R., Castellana E., Ciani E. (2012). Genetic diversity within and among endangered local cattle breeds from Tuscany (Italy). Large Anim. Rev..

[40] Kramarenko A.S., Gladyr E.A., Kramarenko S.S., Pidpala T.V., Strikha L.A., Zinovieva N.A. (2018). Genetic diversity and bottleneck analysis of the Red Steppe cattle based on microsatellite markers. Ukr. J. Ecol..

[41] Sawicka-Zugaj W., Chabuz W., Litwińczuk Z., Kasprzak-Filipek K. (2018). Evaluation of reproductive performance and genetic variation in bulls of the Polish White-Backed breed. Reprod. Domest. Anim..

[42] Brickell J.S., Bourne N., McGowan M.M., Wathes D.C. (2009). Effect of growth and development during the rearing period on the subsequent fertility of nulliparous Holstein-Friesian heifers. Theriogenology.

[43] Gandini G., Maltecca C., Pizzi F., Bagnato A., Rizzi R. (2007). Comparing local and commercial breeds on functional traits and profitability: The case of Reggiana dairy cattle. J. Dairy Sci..

[44] Muller C.J.C., Potgieter J.P., Cloete S.W.P. The Fertility of South African Holstein and Jersey Heifers. Proceedings of the 10th World Congress on Genetics Applied to Livestock Production.

[45] Brzáková M., Zavadilová L., Pribyl J., Pešek P., Kašná E., Kranjčevičová A. (2019). Estimation of genetic parameters for female fertility traits in the czech holstein population. Czech J. Anim. Sci..

[46] Găvan C., Dragan F., Motorga V. (2014). Age of First Calving and Subsequent Fertility and Survival in Holstein Friesian Cattle. Sci. Pap. Anim. Sci. Biotechnol..

[47] Bieber A., Wallenbeck A., Leiber F., Fuerst-Waltl B., Winckler C., Gullstrand P., Walczak J., Wójcik P., Neff A.S. (2019). Production level, fertility, health traits, and longevity in local and commercial dairy breeds under organic production conditions in Austria, Switzerland, Poland, and Sweden. J. Dairy Sci..

[48] Polish Federation of Cattle Breeders and Dairy Farmers (2022). Cattle Assessment and Breeding, Data for 2021. PFHiPM. https://pfhb.pl/fileadmin/user_upload/OCENA/publikacje/publikacje_2022/wyniki_oceny/Wyniki_oceny_za_rok_2021_PFHBiPM_Polska.pdf.

